# Literary Notes

**Published:** 1897-03

**Authors:** 


### Literary Notes.

DR. DEERING J. ROBERTS, chairman of section "B" medical history (Tennessee especially), books, statistics, etc., of the Tennessee Centennial and International Exposition, has issued the following circular: The Centennial and International Exposition, to celebrate the one hundredth year of statehood by the citizens of Tennessee, is now an established fact, and the opening day is fixed for Saturday, May 1st, prox. Already the sum of over $500,000 has been subscribed by the citizens of the state alone, most of which has been very economically and judiciously expended in the erection of large, commodious, elegant and suitable buildings, and in the ornamentation of the grounds. An additional $130,000 has been appropriated by Congress for a special government exhibit. The state legislature now in session will undoubtedly appropriate $100,000 at least, if not more. Many counties in the state are preparing for special exhibits ; and other states and cities are taking active and important steps to have a full representation before the large multitudes of people from all parts of the world, who will visit the capital city of the volunteer state at some time between the 1st of May and the 31st of October next.

A separate and special building for medical and surgical appliances and hygiene will be provided for everything pertaining to these sciences and arts. This building will be specially attractive to the many medical men who will be in attendance from all parts of the world at some time during the season. A dozen or more prominent medical organisations have signified their intention of meeting in Nashville during the centennial, as well as many doctors who are in affiliation with other orders, societies and organisations that will meet here.

The committee in charge of section "B" medical history and literature beg leave to respectfully suggest that you keep a special file or files of your periodical from the first of the current year until the opening day, to be placed in the space allotted, and that you send one or more copies regularly for inspection by the visitors during the progress of the exposition. Should you deem proper to avail yourself of this opportunity of bringing your publication to the notice of our visitors, an early communication to that effect will be appreciated. At a later date full instructions will be given as to when and how this special file and subsequent issues of your publication may be sent.

---

E. B. Treat, medical publisher, of New York, announces that he has in press for early issuance the *International Medical Annual*, being the fifteenth yearly issue of that well-known one-volume reference work. The prospectus shows that the volume will be the result of the labors of upward of forty physicians and surgeons of international reputation, and will present the world's progress in medical science.

The publisher states that he has spared no expense in its production, while the editorial staff has devoted a large amount of time and labor in so condensing the literary matter as to confine the volume within a reasonable size without omitting facts of practical importance.

The value of the work will be greatly enhanced by the thoroughness of illustration; both colored plates and photographic reproductions in black and white will be used wherever helpful in elucidating the text.

It is a work to be commended to all who need a condensed presentation of the literature of the past year.

The volume will contain about 700 pages. The price will be the same as heretofore, $2.75. Full descriptive circular will be sent upon application to the publisher, 5-13 Cooper Union, New York.

---

State Requirements for the Practice of Medicine is the title of a brochure of 85 pages, standard octavo, compiled and issued by Dr. Charles McIntire, of Easton, Pa. This is a guide to the qualifications and methods of procedure to enter upon the practice of medicine in the several states of the union. It will be found invaluable to physicians, teachers, students and especially recent graduates, and can be obtained for the small price of 50 cents enclosed to Dr. McIntire.

We consider it by all odds the most important document that has been issued relating to state license to practise medicine.

---

William R. Warner & Co., pharmaceutical chemists, Philadelphia, have just completed a 300-page pocket medical dictionary, size, 6½ by 4 inches, which will be ready for delivery about March 10, 1897. It is the intention of the Messrs. Warner to offer this dictionary to the medical profession at a figure approximating the cost, or about 75 cents. Advance sheets indicate the type and presswork of the book to be excellent and the definitions simple yet comprehensive.

Orders should be addressed to William R. Warner & Co., 1228 Market street, Philadelphia, Pa.

---

The *Journal of Cutaneous and Urinary Disease* is edited by Drs. James C. Johnston and George K. Swinburne, and published by the Physicians' Publishing Co., at 115 West Eighty-fourth street, New York. We are glad to be able to oblige our contemporary, which is conducted on the most dignified and scientific lines, by giving space to the above notice. It is a journal worthy of the esteem and confidence of the medical profession.

---

The twentieth Year-book of the New York State Reformatory, located at Elmira, is before us. Besides the usual reports of officers, it contains the statistics of inmates, the physician's report and many other interesting data. Under the head of Observations and notes we find much material that will prove of interest to philanthropists, scientists and physicians.

The section on anthropology contains a grouping of nineteen tables, the study of which adds interest to the book. There is no more humane institution than the Elmira Reformatory, whether viewed from the standpoint of economics, morality, philanthropy or Christianity. As usual, this book is well illustrated and handsomely printed.

McKesson & Robbins have published the Physician's Vest-Pocket Formula Book, which will be found very useful to the practitioner. It contains a table of weights and measures, antidotes to poisons, various tables of reference, and a very complete series of tables, showing the composition of foods and alcoholic liquors. These tables should prove valuable to the physician in cases where special attention to dietary is necessary. The book also contains an extended series of notes on some of the new pharmaceutical preparations and a complete list of formulæ of the McKesson & Robbins Gelatine Coated Pills. A copy will be sent free of charge to any of our readers on application to McKesson & Robbins, 91 Fulton street, New York, with mention of the Journal.

---

The *Laryngoscope*, published at St. Louis, has been selected as the official organ, for the year 1897, of the Laryngological Section of the New York Academy of Medicine. This selection, and the great probability of the same journal being chosen by other laryngological, rhinological and otological societies as their official organ, would indicate that the *Laryngoscope* has become what its proprietors stated they intended to make it, *i. e.*, *The American Journal of Record*, for the specialties represented.

---

Ten Cent Journalism.—It is not so very remarkable that certain medical publishers should be able to secure a list of subscribers, when subscriptions are accepted for six months "on trial," for 10 cents! But what must an advertiser think of the employment of such measures to pad a subscription list? Is it not confessedly a weakness on the part of the publisher to admit that he cannot get subscribers unless he accepts them at the paltry price of 20 cents a year? Surely, he must have sample copies "to burn." Shades of the immortal Flint! Has it come to this, that the advertiser must not only pay for the space he occupies, but for the doctors' subscriptions as well? A strange part of it is that so many of the names on these "10 cent" lists should have no initials prefixed Not quite sure, evidently, of the correct names of all these bona-fide 10 cent subscribers. Wonder what the postoffice department would say about a subscription list gained in this manner. The section of postal laws pertaining to the admission of second-class matter in the mails distinctly states that the privilege shall be at once denied whenever it shall be shown that the publication is issued "primarily for advertising purposes, or if the subscription price is nominal." Would you call 20 cents "nominal"?—*American Medical Journalist.*

